# Primary carnitine deficiency: Estimation of prevalence in Chinese population and insights into newborn screening

**DOI:** 10.3389/fgene.2023.1304458

**Published:** 2023-12-06

**Authors:** Xiaoshan Ji, Yanzhuang Ge, Qi Ni, Suhua Xu, Zhongmeng Xiong, Lin Yang, Liyuan Hu, Yun Cao, Yulan Lu, Qiufen Wei, Wenqing Kang, Deyi Zhuang, Wenhao Zhou, Xinran Dong

**Affiliations:** ^1^ Center for Molecular Medicine, Children’s Hospital of Fudan University, National Children’s Medical Center, Shanghai, China; ^2^ Division of Neonatology, National Children’s Medical Center, Children’s Hospital of Fudan University, Shanghai, China; ^3^ Center for Molecular Medicine, Children’s Hospital of Fudan University, National Children’s Medical Center, Institutes of Biomedical Sciences, Fudan University, Shanghai, China; ^4^ Children’s Hospital of Shanghai, Shanghai, China; ^5^ Division of Neonatology, Maternal and Child Health Care Hospital of Guangxi Zhuang Autonomous Region, Nanning, China; ^6^ Division of Neonatology, Children’s Hospital Affiliated to Zhengzhou University, Zhengzhou, China; ^7^ Division of Pediatrics, Xiamen Children’s Hospital, Xiamen, China; ^8^ Guangzhou Women and Children’s Medical Center, Guangzhou Medical University, Guangzhou, China

**Keywords:** primary carnitine deficiency, SLC22A5, prevalence estimation, newborn screening, genotype–phenotype analysis

## Abstract

Primary carnitine deficiency (PCD) caused by pathogenic variants in the solute carrier family 22 member 5 (*SLC22A5*) gene is a rare autosomal recessive disease that results in defective fatty acid oxidation. PCD can be detected through tandem mass spectrometry (MS/MS), but transplacental transport of free carnitine from mothers may cause false negatives or positives during newborn screening (NBS). This study aimed to analyze the genetic characteristics of *SLC22A5* and estimate the prevalence of PCD in the Chinese population, providing useful information for NBS and genetic counseling. We manually curated *SLC22A5* pathogenic or likely pathogenic (P/LP) variants according to the American College of Medical Genetics and Genomics (ACMG) guidelines and identified 128 P/LP variants. Based on the China Neonatal Genomes Project (CNGP), the estimated PCD prevalence was 1:17,456, which was higher than that in other populations. The genotype–phenotype association analysis showed that patients carrying homozygous c.760C>T and c.844C>T were more likely to present cardiomyopathy, whereas those carrying homozygous c.1400C>G were more likely to be asymptomatic (all *p*-values < 0.05). We found that there was no significant difference in initial C0 concentrations between patients and carriers, but there was a significant difference in the second-tier screening of C0 concentration between them (*p*-value < 0.05). We established a cost-effective variant panel containing 10 high-frequency sites and developed a screening algorithm incorporating gene panels with MS/MS, which could rescue one more patient who was undetected from MS/MS. In conclusion, the prevalence of PCD in the Chinese population is relatively high. The combination of conventional NBS with genetic sequencing is suggested for early diagnosis of PCD.

## 1 Introduction

Primary carnitine deficiency (PCD, OMIM #212140) is an autosomal recessive carnitine transport defect caused by biallelic pathogenic variants in the solute carrier family 22 member 5 (*SLC22A5*) gene, which encodes organic cation/carnitine transporter type 2 (OCTN2) (Nezu et al., 1999; Tang et al., 1999; Picard, 2022). OCTN2 is strongly expressed in the kidney, skeletal muscle, heart, and placenta (Tamai et al., 1998), and its defect results in urinary carnitine wasting and low serum carnitine levels, leading to defective fatty acid oxidation. The clinical manifestations of PCD can vary widely with respect to the age of onset, involved organs, and severity of symptoms. It encompasses a broad clinical spectrum including metabolic decompensation, cardiomyopathy, fatigability, or absence of symptoms. Due to newborn screening (NBS), many asymptomatic mothers have been given the diagnosis of PCD (Magoulas and El-Hattab, 2012; Almannai et al., 2019). Recent research has shown a high correlation between sudden death and untreated PCD, particularly in females (Rasmussen et al., 2020). As a result, patients with PCD are at risk for sudden death throughout their lifetime. Conversely, early detection and carnitine therapy can prevent metabolic decompensation and death, and the long-term prognosis is good.

The prevalence of PCD varies in different countries: 1:20,000–1:70,000 in the United States (Gallant et al., 2012), 1:40,000 in Japan (Shibata et al., 2018), and 1:120,000 in Australia (Webster et al., 2003). The highest prevalence is 1:300 in the Faroe Islands (Rasmussen et al., 2014). In China, the prevalence of PCD varies among diverse regions, ranging from 1:8,938 to 1:45,000 (Lin et al., 2020; Yang et al., 2021). The overall PCD prevalence of Chinese population remains elusive.

There is a correlation between genotype and carnitine levels. It has been reported that patients with a homozygous nonsense mutation of *SLC22A5* had lower carnitine levels than those with missense mutations or heterozygous nonsense mutations. In addition, the closer the truncation is to the C-terminal domain, the lower the level of carnitine is (Rose et al., 2012; Shibbani et al., 2014). However, carnitine levels do not indicate severity of phenotypic presentation. No clear correlation could be established between the genotype and severity of clinical presentation or age of onset, suggesting that environmental factors such as drugs, fasting, or infection were responsible for the wide variability in phenotypic expression in PCD (Lamhonwah et al., 2002; Ying, 2023).

With the advent of expanded NBS, infants with PCD can be identified based on low free carnitine (C0) levels using tandem mass spectrometry (MS/MS). However, carnitine can transfer from the mother to the fetus through the placenta during pregnancy, which may lead to a false C0 level affected by the maternal concentration. In addition, secondary carnitine deficiency can be caused by a variety of reasons, such as malnutrition, malabsorption, and several inherited metabolic disorders including fatty acid oxidation disorders and organic acidemias (Almannai et al., 2019). Therefore, the definite diagnosis of PCD relies on the genetic analysis of *SLC22A5* gene or the measurement of carnitine transport activity in fibroblasts. The China Neonatal Genomes Project (CNGP) includes 98 hospitals, spanning the entire country (Wu et al., 2021; Xinran Dong et al., 2022). In this study, we assessed 278 *SLC22A5* variants recruited from the CNGP and public database, and identified 128 pathogenic/likely pathogenic (P/LP) variants. We found that different populations had different pathogenic hotspots in *SLC22A5*. We estimated the prevalence of PCD in the Chinese population, reviewed the published cases to further understand the genotype–phenotype correlation, and discussed a suitable screening algorithm for PCD diagnosis in China.

## 2 Materials and methods

### 2.1 Collection of Chinese population data

This study was approved by the Ethics Committee of Children’s Hospital of Fudan University (CHFudanU_NNICU11). Neonates in the CCGT database who underwent genetic tests from August 2016 to December 2021 were all included. The detailed processing steps were described in our previous study (Xiao et al., 2021; Xiao et al., 2022). In brief, counseling was conducted, and informed consents were obtained from the parents of patients. Each individual underwent whole-exome sequencing (WES) or clinical exome sequencing (CES), with both covering the exon region and exon–intron splicing junction region (deep intron to 15bp) of *SLC22A5* gene. Both tests were sequenced on the Illumina HiSeq X10 with 150 bp pair-end. Infants with genetic positive results were followed up in the 3rd month after hospital discharge and recalled for examination of the MS/MS test, liver ultrasonography, and heart color ultrasound. Diagnostic decisions were made based on the confirmatory test results (C0 in the lower limit, which was 10 μmol/L in our laboratory).

### 2.2 Literature search of PCD-related studies

PubMed and Web of Science were searched using the following terms: “primary carnitine deficiency,” “carnitine transport defect,” “carnitine uptake defect,” “*SLC22A5* mutation,” “*SLC22A5* variant,” “*OCTN2* mutation,” and “*OCTN2* variant” between 1999 (pathogenic variant was first described) and December 2022 (Nezu et al., 1999). Studies meeting the following inclusion criteria were selected: 1) case reports in which the nomenclature of mutation sites meets the requirements of HGVS (den Dunnen et al., 2016), 2) the sites of the case reports were evaluated as P/LP according to the American College of Medical Genetics and Genomics (ACMG) guidelines, and 3) the study was included in SCI (represents high-quality literature). Exclusion criteria included 1) lack of information of mutation sites or the nomenclature of mutation sites, 2) lack of clinical information, 3) complex cases with more than two P/LP variants, and 4) repeated cases. According to those criteria, a total of 881 articles were found, of which 53 were finally included in this study and 293 published articles of PCD patients were collected for genotype–phenotype association studies.

### 2.3 Curation of P/LP variants in *SLC22A5* gene

We included reported pathogenic variants of the *SLC22A5* gene from ClinVar (level P or LP), HGMD (level DM or DM?), and PCD-related literature mentioned earlier. No new variants were reported in the CNGP database. These variants were curated by two clinical geneticists back-to-back according to the ACMG guidelines. After manual inspection, 128 out of 281 variants were curated at the P/LP level.

### 2.4 Collection of public *SLC22A5* variant frequency

ChinaMAP was introduced as an external database of the Chinese population. It is based on cohort studies across diverse regions and ethnic groups with metabolic phenotypic data in China, and analysis of the whole-genome sequencing data in 10,588 healthy individuals (Cao et al., 2020).

The allele frequency (AF) of *SLC22A5* gene in other populations was available from the gnomAD database. AFR, AMR, ASJ, FIN, NFE, SAS, and EAS populations in gnomAD were included.

### 2.5 Estimation of PCD prevalence in the Chinese population

We estimate the PCD prevalence using three strategies as described in our previous studies (Ni et al., 2022). Samples identified or suspected of PCD were excluded, and PCD prevalence was estimated using three methods. Method 1 was based on the carrier frequency of individuals, which was calculated by the Hardy–Weinberg principle. Method 2 was based on the principle of permutation and combination in mathematics. In this strategy, the hypothesis is to calculate the probability of an affected child by randomly choosing a male individual carrying a P/LP variant in the *SLC22A5* gene and a female individual also carrying a P/LP variant in the *SLC22A5* gene. Method 3 was based on the Bayesian framework with the gnomAD allele count dataset, where 95% confidence interval could be estimated (Schrodi et al., 2015).

### 2.6 Data acquisition and processing for the study of the genotype–phenotype relationship

We collected the genetic information and clinical characteristics of PCD patients from PCD-related literature mentioned earlier to study genotype–phenotype relationships. After manual inspection, 293 out of 447 PCD patients were included. Here, 16 common manifestations were inferred from GeneReviews. The variants were further grouped by their site, mutation type, and zygosity. Fisher’s exact test and Chi-squared test were applied to testify whether one phenotype was over-represented in one type of mutations compared with others.

### 2.7 Statistical analysis

All statistical analyses were performed by R version 4.0.3. Chi-squared test (λ2.test) was used by default. When the conditions were not met, Fisher’s exact test was used; *p*-values were adjusted by the “fdr” strategy for multiple tests.

## 3 Results

### 3.1 Curation of *SLC22A5* pathogenic variants and allele frequency analysis

**FIGURE 1:**
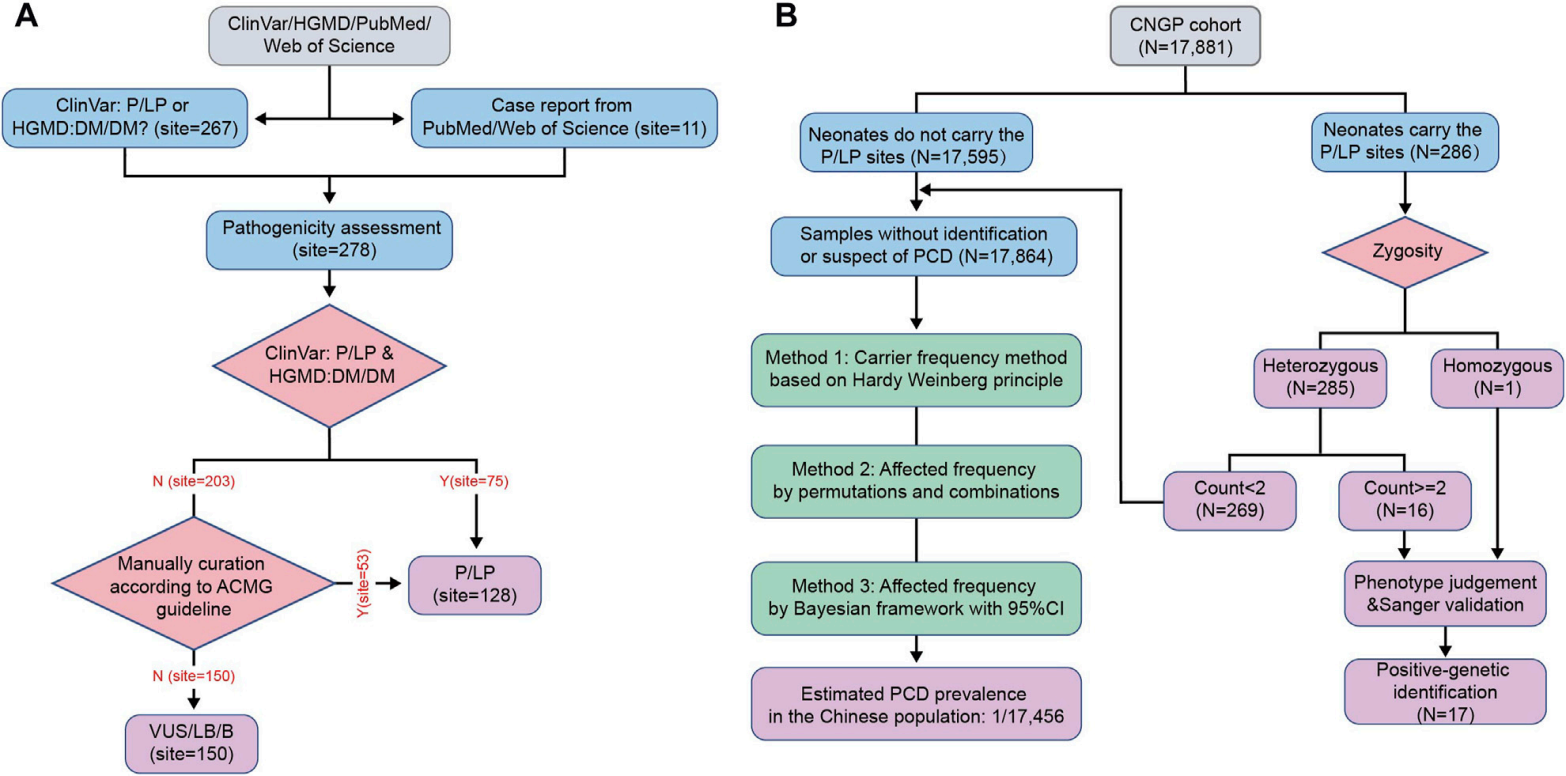
Flow diagram of the study. **(A)** Workflow for curation of pathogenic variants in *SLC22A5* gene. **(B)** Workflow for PCD identification and estimation of PCD prevalence in the Chinese population. HGMD, Human Gene Mutation Database; P, pathogenic; LP, likely pathogenic; DM, disease-causing mutation; ACMG, American College of Medical Genetics and Genomics; VUS, versus uncertain significance; LB, likely benign; B, benign; CNGP, China Neonatal Genomes Project; PCD, primary carnitine deficiency; 95% CI, 95% confidence interval.

**FIGURE 2:**
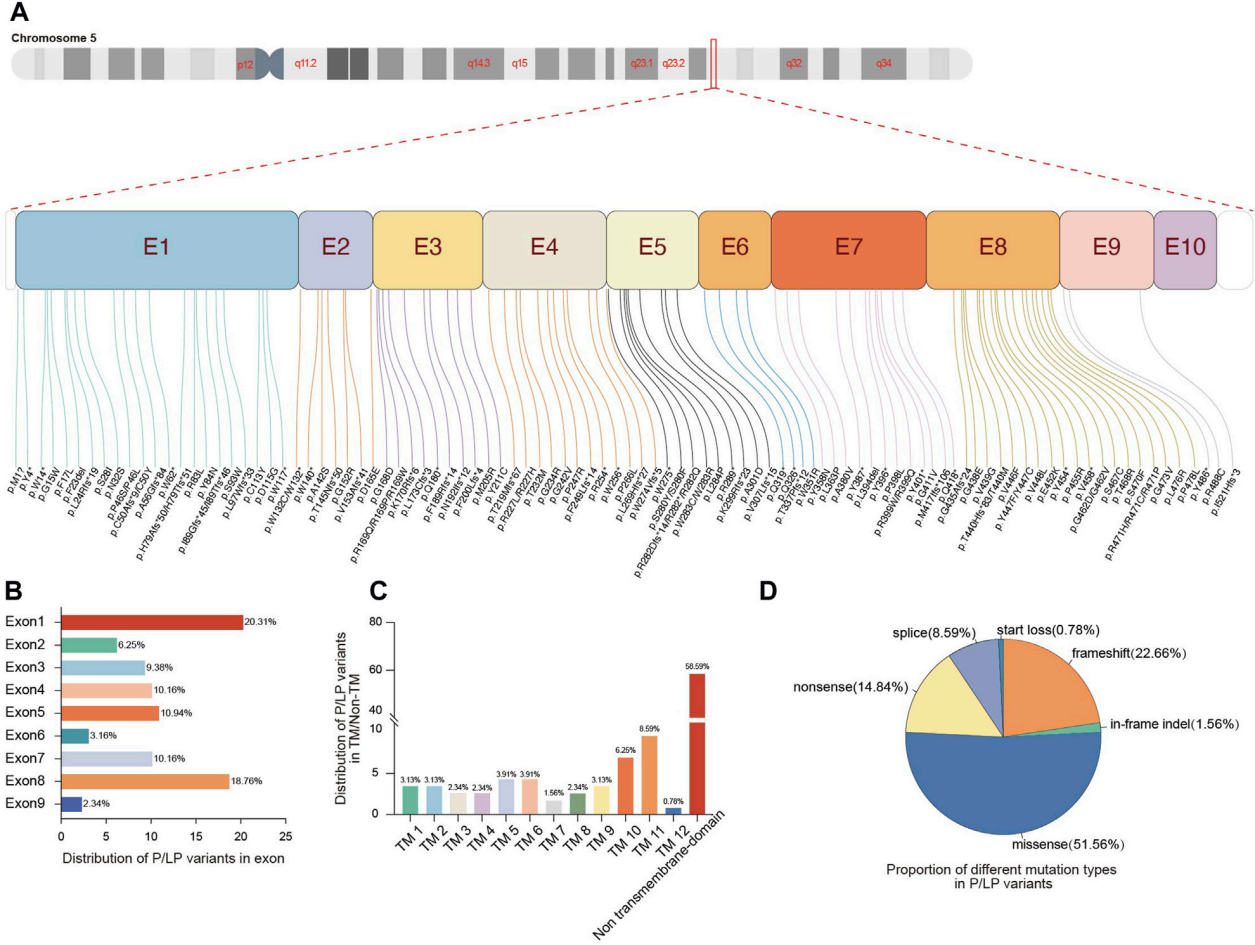
Genetic spectrum of pathogenic variants in the SLC22A5 gene. **(A)** Distribution of P/LP variants in the 10 exons of *SLC22A5* gene. **(B)** Proportion of different exons in P/LP variants. **(C)** Proportion of different functional domains in P/LP variants. **(D)** Proportion of different mutation types in P/LP variants. E, exon; TM, transmembrane; P, pathogenic; LP, likely pathogenic.

After pathogenicity assessment of 278 *SLC22A5* variants collected from the CNGP, Human Gene Mutation Database (HGMD), ClinVar, Web of Science, and PubMed, 128 pathogenic or likely pathogenic (P/LP) variants were identified (Figure 1A; Supplementary Table S1). All P/LP variants were scattered on exons (91.40%, 117/128) and flanking introns (8.59%, 11/128) (Figure 2A). The most common affected exons were exon 1 (20.31%, 26/128) and exon 8 (18.75%, 24/128) (Figure 2B), and the most common affected functional domain was the non-transmembrane domain (58.59%, 74/128) (Figure 2C). The mutation types included missense (51.56%, 66/128), frameshift (22.66%, 29/128), nonsense (14.84%, 19/128), splicing (8.59%, 11/128), in-frame indel (1.56%, 2/128), and start loss (0.78%, 1/128) variants (Figure 2D).

**FIGURE 3:**
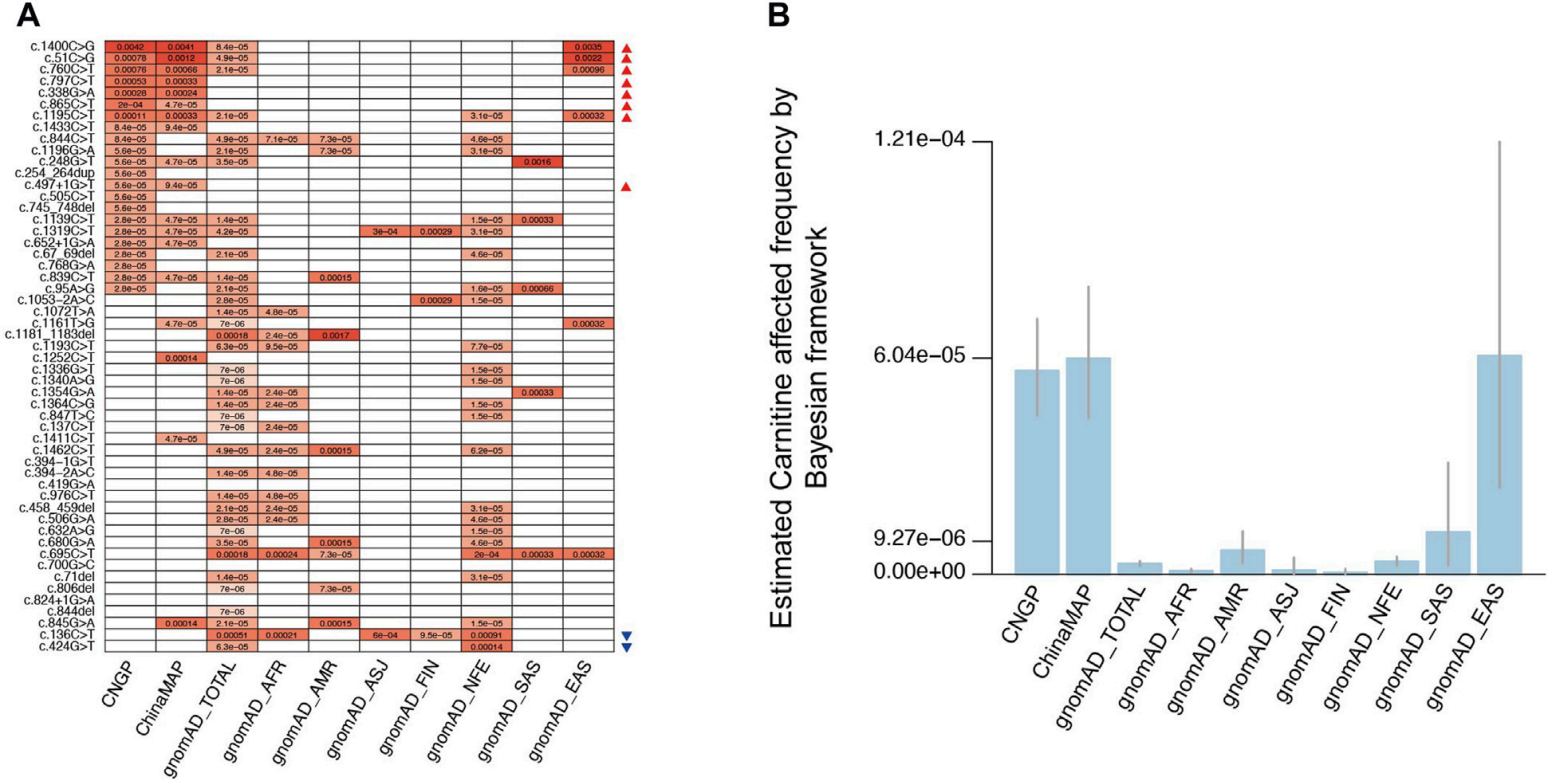
Allele frequency and estimated PCD frequency in different populations. **(A)** Allele frequency comparison for 53 pathogenic variants in different populations. **(B)** Estimated PCD affected the frequency of different populations by the Bayesian framework. CNGP, China Neonatal Genomes Project; AFR, African American; ASJ, Ashkenazi Jewish; NFE, non-Finland European; FIN, Finnish in Finland; AMR, admixed American; SAS, South Asian; EAS, East Asian. **(A)** Red triangles indicate variants with higher AF in the Chinese population than in non-East Asian populations, and blue triangles indicate variants with a significantly lower AF value in the Chinese population. **(B)** We employed scientific notation to express numbers, whereas in the main text, we present them as fractions. It is important to note that these representations are equivalent, that is, 9.27e-06 is equivalent to 1:107,875, 6.04e-05 is equivalent to 1:16,556, and 1.21e-04 is equivalent to 1:8,264.

Among the 128 P/LP variants, 53 had documented AF in general populations from public databases (Figure 3A; Supplementary Table S2). Altogether, eight P/LP variants (c.51C>G, c.338G>A, c.760C>T, c.797C>T, c.865C>T, c.1195C>T, c.1400C>G, and c.497+1G>T) had significantly higher AF in the Chinese population than in non-East Asian populations, whereas two variants (c.136C>T and c.424G>T) had significantly lower AF value in the Chinese population. The most frequent P/LP site in the gnomAD-TOTAL (all populations in gnomAD) was c.136C>T (1:1,965 or 0.00051), which was also the most frequent site in Ashkenazi Jewish (ASJ, 1:1,661 or 0.00060) and non-Finland European populations (NFE, 1:1,094 or 0.00091), consistent with the previous study (Magoulas and El-Hattab, 2012). The most common variant site in the Chinese population was c.1400C>G (ChinaMAP: 1:243 or 0.0041, CNGP: 1:239 or 0.0042), and it has not been reported in non-East Asian populations. The other high-frequency pathogenic sites in the Chinese population were c.51C>G (ChinaMAP: 1:833 or 0.0012, CNGP: 1:1,275 or 0.00078) and c.760C>T (ChinaMAP: 1:1,512 or 0.00066, CNGP: 1:1,322 or 0.00076), both of which have not been reported in non-East Asian populations. These results suggested that the Chinese population had special characteristics in *SLC22A5* variants.

### 3.2 Estimation of PCD prevalence in the Chinese population

In the CNGP cohort, we enrolled a total of 17,881 neonates. To estimate PCD prevalence, we excluded neonatal patients identified or suspected of PCD, leaving 17,864 (7,333 males and 10,531 females) neonates (Figure 1B). The number of individuals carrying P/LP *SLC22A5* variants was 269 (144 males and 125 females). Based on the carrier frequency, the estimated PCD prevalence was 1:17,641 in the CNGP. By the permutation and combination method, the estimated prevalence was 1:17,161. By the Bayesian framework, the estimated prevalence of PCD was 1:17,576 (95% confidence interval: 1:22,551–1:14,001). In general, the estimated prevalence of PCD in the Chinese population is 1:17,456 by averaging all the above results (Table 1).

**TABLE 1:** PCD prevalence estimation in the CNGP cohort with estimated affected frequency by three methods.

	CNGP cohort
Total number	17,864
Gender (female/male)	7,333/10,531
Carrier with P/LP variants (female/male)	125/144
Carrier frequency	1:66
Method 1: carrier frequency	1:17,641
Method 2: permutation and combination	1:17,161
Method 3: Bayesian framework (95% CI)	1:17,576 (1:22,551–1:14,001)
Average	1:17,456

P/LP, pathogenic or likely pathogenic; CNGP, China Neonatal Genomes Project; CI, confidence interval.

For the result of PCD prevalence estimation by the Bayesian framework in other gnomAD populations, the prevalence is 1:1,134,461 (95% confidence interval: 1:2,300,325–1:650,711) in African American (AFR) population, 1:148,320 (95% confidence interval: 1:314,155–1:82,852) in admixed American (AMR) population, 1:921,025 (95% confidence interval: 1:28,837,593–1:211,888) in ASJ population, 1:1,964,440 (95% confidence interval: 1:13,880,667–1:645,273) in Finnish in Finland (FIN) population, 1:282,613 (95% confidence interval: 1:413,709–1:202,688) in NFE population, 1:84,707 (95% confidence interval: 1:404,329–1:31,975) in South Asian (SAS) population, and 1:16,375 (95% confidence interval: 1:41,410–1:8,272) in East Asian (EAS) population (Figure 3B). Based on these results, we found the PCD prevalence in the Chinese population was much higher than that in other populations, especially higher than that in FIN population.

### 3.3 Analysis of the genotype–phenotype relationship in PCD patients

We collected genetic and clinical information of 293 PCD patients from the literature review (Supplementary Table S3). For infants and children, cardiomyopathy (71/194, 36.5%) was the dominant phenotype. Other common phenotypes included hypoglycemia (32/194, 16.5%), hyperammonemia (22/194, 11.3%), hepatomegaly (33/194, 17.0%), and elevated ALT (31/194, 15.9%). Most adult patients were asymptomatic or experienced only fatigue (88/97, 90.7%). We analyzed the relationship between the phenotype and variant site, mutation type, and zygosity. For each variant–phenotype analysis, we found that patients carrying homozygous c.760C>T and c.844C>T were more likely to present cardiomyopathy than those with a combination of other variant sites (OR = 10.5 in c.760C>T and OR = +∞ in c.844C>T, both *p*-value < 0.05), whereas those carrying homozygous c.1400C>G were more likely to be asymptomatic than those without these variants (OR = +∞, *p*-value < 0.05; Supplementary Table S4). Mutation types of all variants in the 293 PCD patients were classified into the following: missense, frameshift, nonsense, splicing, in-frame indel, and start loss variants. Two missense variants were the most common mutation type combination in PCD patients (177/293, 60.4%), then were two nonsense variants (43/293, 14.6%), and a combination of one nonsense variant and one missense variant (34/293, 11.6%). For mutation type–phenotype analysis, we found that patients carrying two frameshift variants and patients carrying two nonsense variants were more likely to present cardiomyopathy (OR = 10.8 in frameshift variants and OR = 7.1 in nonsense variants, both *p*-values < 0.05), whereas patients carrying two missense variants were more likely to be asymptomatic (OR = 3.8, *p*-value < 0.05; Supplementary Table S5). For zygosity–phenotype analysis, patients carrying compound heterozygous pathogenic variants were more likely to be asymptomatic (OR = 4.2, *p*-value < 0.05), whereas patients carrying homozygous pathogenic variants were more likely to present cardiomyopathy (OR = 4.0, *p*-value < 0.05) and cardiac failure (OR = 13.7, *p*-value < 0.05; Supplementary Table S6).

### 3.4 Patients in CNGP cohort and discussion of a screening algorithm

Seventeen neonates were diagnosed as genetically positive cases of PCD in the CNGP cohort, including 16 compound heterozygous neonates (heterozygous mutation in each allele) and one homozygous. Fifteen of them were diagnosed through NBS, whereas two patients had normal initial C0, suggesting that a proportion of cases (11.7%, 2/17) were missed by conventional NBS.

**FIGURE 4:**
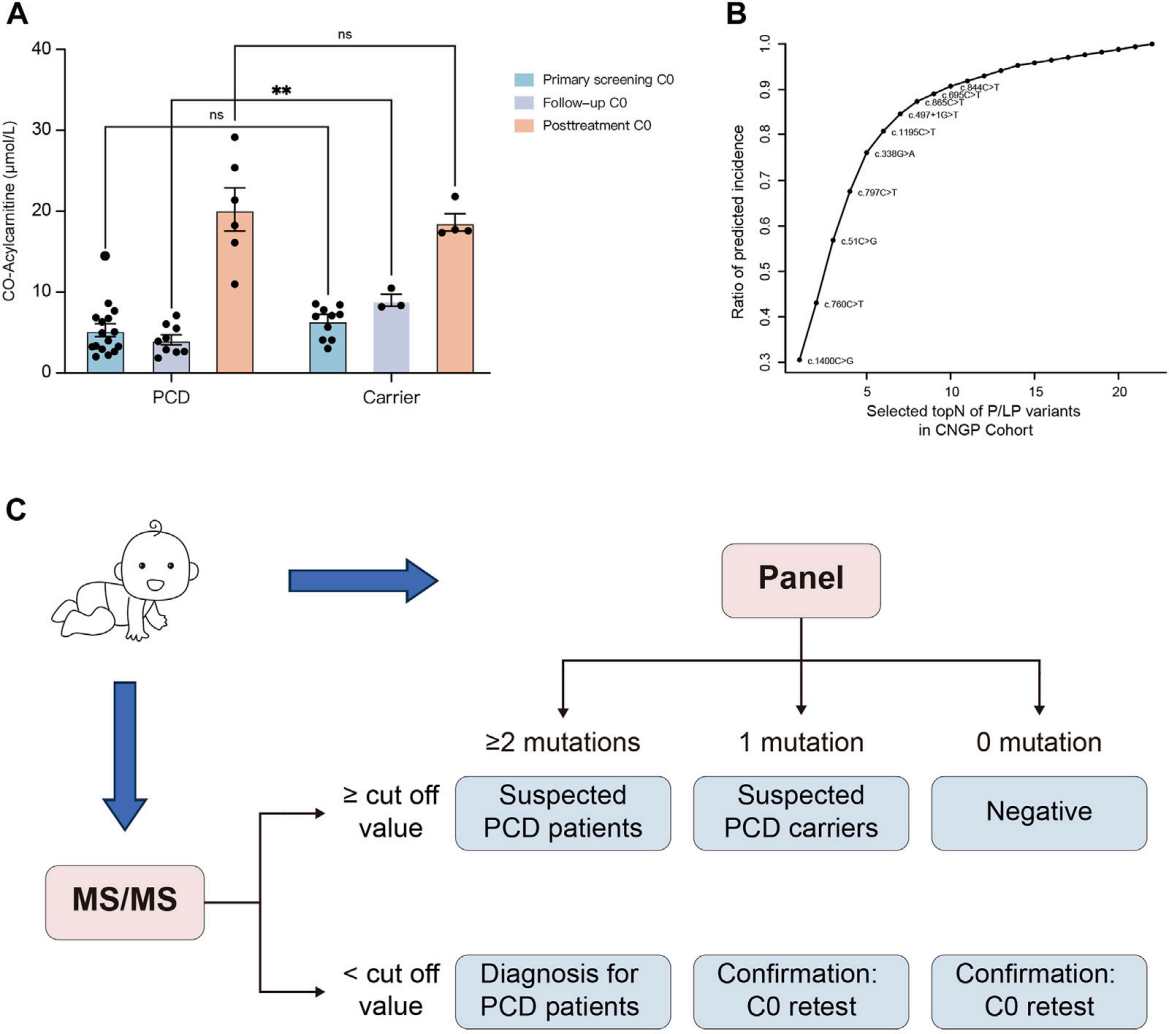
Establishment of the screening algorithm for the Chinese population. **(A)** Primary screening C0, follow-up C0, and post-treatment C0 concentrations in dried blood spots from carriers and PCD patients. **(B)** Estimated incidence based on P/LP variants from the CNGP cohort. Rapid detection of the top 10 high-frequency sites (c.1400C>G, c.760C>T, c.51C>G, c.797C>T, c.338G>A, c.1195C>T, c.497 + 1G>T, c.865C>T, c.696C>T, and c.844C>T) may cover approximately 90% of Chinese pediatric patients. **(C)** Designed screening algorithm for PCD in the Chinese population. Primary screening is performed at 48–72 h after birth; if C0 is higher than the cut-off value (10 μmol/L in our laboratory), newborns with two, one, and no *SLC22A5* mutations can be suspected as patients, carriers, and negatives, respectively. If C0 is lower than the cut-off value, newborns with more than two mutations can be diagnosed as patients. In other cases, a duplicate retest on the birth dried blood spot is performed. PCD, primary carnitine deficiency; CNGP, China Neonatal Genomes Project; MS/MS, tandem mass spectrometry.

The initial NBS results were available in 72 heterozygote carriers, and 10 of them had low C0 levels (Supplementary Table S4). During the follow-up period, one carrier developed growth retardation and the remaining carriers were asymptomatic. In comparison, the initial C0 concentrations in neonatal carriers (*n* = 10) and patients (*n* = 17) with low C0 levels were 6.47 and 5.27 μmol/L, respectively (Figure 4A). Without carnitine therapy, the second-tier screening of C0 concentrations in carriers (*n* = 3) increased to 9.01 μmol/L, whereas the C0 concentrations in patients (*n* = 9) decreased to 4.28 μmol/L. After L-carnitine supplementation, the C0 concentrations in carriers (*n* = 4) and patients (*n* = 6) increased to 18.61 and 20.21 μmol/L, respectively.

To establish a cost-effective variant panel for PCD diagnosis, we identified 10 high-frequency sites, namely, c.1400C>G, c.760C>T, c.51C>G, c.797C>T, c.338G>A, c.1195C>T, c.497 + 1G>T, c.865C>T, c.696C>T, and c.844C>T (Figure 4B). These variants were the top 10 variants in the CNGP cohort and covered approximately 90% of Chinese patients. Coupling the screening panel on initial MS/MS, we developed a screening algorithm for PCD (Figure 4C). The algorithm could identify 15 (15/17, 88.2%) newborns as positive in the first-tier test, whereas another two (2/17, 11.8%) newborns would be identified as suspected carriers and needed second-tier testing and follow-up. One patient undetected from MS/MS could be benefitted from the implementation of gene panels as a first-tier screening test.

## 4 Discussion

The *SLC22A5* gene is located on the chromosome 5q31 and contains 10 exons, encoding 557 amino acid polypeptides (Saito et al., 2002). In this study, we curated 281 *SLC22A5* variants recruited from the CNGP and public databases and identified 128 P/LP variants. A few ethnic-specific variants have been reported in several populations. For instance, c.95A>G is the founder variant of the Faroe Islands (Rasmussen et al., 2014), c.136C>T is the most frequent mutation in the United States of America (Frigeni et al., 2017), and c.396C>T and c.1400C>G are common variants in Japan (Koizumi et al., 1999). In our study, we found that c.1400C>G was the most prevalent variant in the Chinese population, followed by c.51C>G and c.760C>T, which is consistent with previous studies (Lin et al., 2019; Lin et al., 2020). Some studies indicated that c.760C>T was the most frequent variant in Chinese patients, but the sample size was relatively limited (Han et al., 2014; Lin et al., 2019; Lin et al., 2022). It has been reported that the variants presented different geographic distributions in China (Lin et al., 2022). For instance, c.760C>T, a severe mutation with very low residual OCTN2 transporter activity, was common in southern China but rarely detected in northern China (Lin et al., 2019; Yang et al., 2020; Zhang et al., 2021; Lin et al., 2022). In contrast, c.1400C>G with a residual function that may result in a mild phenotype was common in both southern and northern China (Lin et al., 2020; Yang et al., 2020; Zhang et al., 2021; Lin et al., 2022).

PCD is the most common fatty acid metabolic disorder in China. Recruiting newborns from 98 hospitals across different regions, this study estimated that the prevalence of PCD in the Chinese population was 1:17,456, which is similar to the prevalence recently reported in Ningbo (1:16,595) (Yang et al., 2021), Guangzhou (1:13,345) (Huang et al., 2020), and Quanzhou (1:11,189) (Lin et al., 2022). The PCD prevalence in the Chinese population is higher than that in other populations, especially much higher than that in Caucasian populations.

The genotype–phenotype correlation suggests that frameshift and nonsense variants are more likely to be associated with cardiomyopathy, which is the main phenotype of PCD, whereas missense variants are more common in asymptomatic individuals. This is consistent with previous studies demonstrating that nonsense and frameshift variants in *SLC22A5* are typically associated with lower carnitine transport, whereas missense variants may result in proteins with retained residual carnitine transport activity (Rose et al., 2012; Shibbani et al., 2014). As for phenotype–variant site correlations, c.760C>T and c.844C>T were identified to be related with cardiomyopathy, and c.1400C>G was related with asymptomatic individuals. Our results confirmed the previous conclusion and gave a clue to the genotype–phenotype association of PCD.

PCD has been included in the NBS plan in China based on MS/MS, which is critical for PCD diagnosis and cannot be replaced. However, the current NBS faces challenges because C0 in days 2–3 has poor sensitivity and a positive predictive value (Wilson et al., 2019). As carnitine can be transported through the placenta, newborns with PCD can have a carnitine supply from their mother, which causes false-negative results. On the other hand, babies born to mothers with PCD can have a low free C0 level, which causes false-positive results. WES and genome sequencing are promising candidates for the genomic sequencing test, but their use as a universal screening test in clinical applications is hindered by their high cost. As PCD is a disorder with relatively high prevalence in China and can be fatal if left untreated, we designed a cost-effective variant panel containing 10 high-frequency sites. Coupling the screening panel on initial MS/MS as a first-tier screening, we established a screening algorithm for PCD, which could identify one more patient undetected by MS/MS. Incorporating gene panels with biochemical NBS is a low-cost approach and could largely reduce the time and expenditure from positive screen to case closure. However, it is noteworthy that the improvement in sensitivity comes at the expense of increased carrier identification. Newborns with only one variant detected need long-term follow-up, and further genetic analysis is required when they have persistently low C0 levels. Large-scale studies are needed to optimize the workflow and to evaluate the cost-effectiveness of this screening approach.

There are some limitations to note about our study. First, the main criterion for inclusion was the identification of P/LP variants in the *SLC22A5* gene. Therefore, the false-positive rate of conventional NBS is unclear. Second, as PCD has been included in NBS in many countries, the patients accepted carnitine supplementation before the phenotypes occurred, which was good for patients but introduced many missing values in the genotype–phenotype analyses. Third, most published cases only reported the chief symptoms and did not mention whether the patients had other phenotypes, resulting in non-significant results. More comprehensive records of patients would help to clarify the genotype–phenotype relationship of PCD.

## 5 Conclusion

The prevalence of PCD is higher in the Chinese population than in Caucasian populations, and c.1400C>G, c.51C>G, and c.760C > T are hotspots of *SLC22A5* in the Chinese population. Frameshift and nonsense variants are associated with cardiomyopathy, whereas missense variants are more common in asymptomatic individuals. The combination of conventional NBS with genetic sequencing is suggested for early diagnosis of PCD.

## Data Availability

The datasets presented in this article are not readily available because the original data contains patients individual and private information. Requests to access the datasets should be directed to PICOTEES framework, a privacy-preserving online service of phenotype exploration for genetic-diagnostic variants (https://birthdefectlab.cn:3000/), or send an email to xrdong@fudan.edu.cn.

## Supplementary material

SUPPLEMENTARY TABLE S1 Manually curated *SLC22A5* variants’ pathogenicity.

SUPPLEMENTARY TABLE S2 Allele frequency for top 53 pathogenic variants to different populations.

SUPPLEMENTARY TABLE S3 Summary of genotype–phenotype information of 293 PCD patients.

SUPPLEMENTARY TABLE S4 Relationship between variant site and phenotype.

SUPPLEMENTARY TABLE S5 Relationship between mutation type and phenotype.

SUPPLEMENTARY TABLE S6 Relationship between zygosity and phenotype.

SUPPLEMENTARY TABLE S7 Genetic testing and C0 results for PCD patients in the CNGP cohort.

SUPPLEMENTARY TABLE S8 Genetic testing and C0 results for PCD carriers with low initial C0.
